# Gene therapy for colorectal cancer using adenovirus-mediated full-length antibody, cetuximab

**DOI:** 10.18632/oncotarget.8596

**Published:** 2016-04-05

**Authors:** Man Xing, Xiang Wang, Yudan Chi, Dongming Zhou

**Affiliations:** ^1^ Vaccine Research Center, Key Laboratory of Molecular Virology and Immunology, Institut Pasteur of Shanghai, Chinese Academy of Science, Shanghai, China

**Keywords:** EGFR, cetuximab, adenovirus, colorectal cancer, gene therapy

## Abstract

Cetuximab is a chimeric monoclonal antibody, approved to treat patients with metastatic colorectal cancer (mCRC), head and neck squamous cell carcinoma (HNSCC), non-small-cell lung cancer (NSCLC) for years. It functions by blocking the epidermal growth factor receptor (EGFR) from receiving signals or interacting with other proteins. Although the demand for cetuximab for the treatment of cancer patients in clinics is increasing, the complicated techniques involved and its high cost limit its wide applications. Here, a new, cheaper form of cetuximab was generated for cancer gene therapy. This was achieved by cloning the full-length cetuximab antibody into two serotypes of adenoviral vectors, termed as AdC68-CTB and Hu5-CTB. *In vivo* studies showed that a single dose of AdC68-CTB or Hu5-CTB induced sustained cetuximab expression and dramatically suppressed tumor growth in NCI-H508– or DiFi-inoculated nude mice. In conclusion, gene therapy using adenovirus expressing full-length cetuximab could be a novel alternative method for the effective treatment of colorectal cancer.

## INTRODUCTION

EGFR is a transmembrane glycoprotein that plays crucial roles in regulating cell proliferation, migration, invasion, adhesion, differentiation and survival [1–3]. The autonomous and dysregulated activation of EGFR is implicated in most cancers [4]. Furthermore, EGFR is overexpressed in a variety of human cancers, such as mCRC, HNSCC, NSCLC, pancreatic cancer, glioblastoma and ovarian carcinoma [5–10]. Given these reasons, EGFR was proposed as an attractive target for anticancer therapy.

Cetuximab is a murine-human chimeric anti-EGFR monoclonal antibody that contains the human lgG1 constant region [11]. The Food and Drugs Administration (FDA) has approved cetuximab to treat mCRC and HNSCC alone or jointly [12, 13]. Cetuximab competitively binds to the extracellular domain of EGFR with higher affinity than other endogenous ligands, such as the epidermal growth factor (EGF) and the transforming growth factor alpha (TGFα) [14, 15]. The binding of cetuximab to EFGR blocks the activation of receptor tyrosine kinase and the downstream signaling pathways, including the RAS-RAF-MEK-MAPK pathway and the PI3K-Akt pathway. The former controls gene transcription, cell cycle progression and cell proliferation, and the latter triggers a series of anti-apoptotic and pro-survival signals [1, 16]. Furthermore, cetuximab down-regulates cell surface EGFR by internalization [13, 17]. It also elicits host antitumor immune responses, including antibody-dependent cellular cytotoxicity (ADCC) and complement-mediated cytotoxicity (CMC) [18, 19].

The associated complicated techniques and high cost limit the wide applications of cetuximab, but its demand is still increasing. Production of the therapeutic antibody is a highly complex biotechnological process. Furthermore, sufficient expression and purity still exist as manufacturing challenges. In pharmacokinetic studies, the mean half-life of cetuximab is approximately 10 days due to its chimeric structure [20]. Patients therefore require a weekly high dose of Erbitux to maintain an effective serum antibody concentration [21]. However, frequent antibody administrations and impurities in the antibody inevitably cause some side effects [22, 23]. Accordingly, *in vivo* antibody gene therapy, which can produce a high-concentration of antibody free from impurities and reduce the side effects and cost, is considered one of the best candidates for long-term therapy [24, 25].

Adenoviruses have become the most commonly used gene therapy vector, considering their high transduction efficiency, broad cell tropism, high gene expression, and mature production technology [26–29]. Adenovirus-mediated gene therapies have typically adopted human serotype 5 (Hu5), however its efficiency is dampened by prevalence of neutralizing antibodies among populations [30, 31]. We developed replication-defective recombinant adenovirus based on the chimpanzee serotype 68 (AdC68) or Hu5, expressing the full-length cetuximab antibody. As AdC68 has comparable excellent expression of foreign genes to Hu5, and lacks neutralizing B-cell epitopes cross-reacting with common human serotypes [32], we reasoned that a therapeutic antibody based on AdC68 is more suitable for cancer therapy in humans. Here, we evaluated the efficacy of adenovirus-mediated anti-EGFR (Ad-anti-EGFR) antibodies against colorectal cancer in mice.

## RESULTS

### Recombinant adenovirus construction

**Figure 1:**
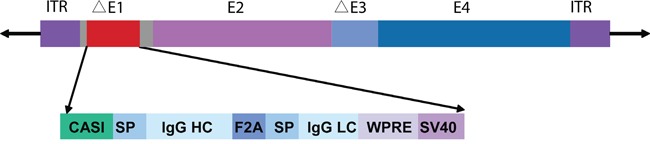
Full-length cetuximab antibody expression cassette Schematic illustration of adenoviral vector with the expression cassette inserted at the E1 region. Antibody light and heavy chains, with separate signal peptides, are linked by F2A. The CASI promoter contains a portion of the CMV enhancer, a portion of the chicken β-actin promoter, and a portion of the UBC enhancer. HC, heavy chain; LC, light chain; SP, signal peptide; WPRE, woodchuck hepatitis virus posttranscriptional regulatory element.

Erbitux (cetuximab; Merck Serono, Rockland, MA), the commercial monoclonal antibody against EGFR, was used as a positive control in our studies. E1- and E3-deleted adenoviral recombinants of Hu5 and AdC68 were developed to express the full-length cetuximab, driven from CASI promoter composed of the cytomegalovirus immediate early promoter (CMV), chimeric chicken-β-actin (CAG), and ubiquitin C (UBC) enhancer region. The light-chain and heavy-chain with separate signal peptides were linked with F2A to constitute the antibody expression cassette that ended with SV40 late poly (A). The woodchuck hepatitis virus posttranscriptional regulatory element (WPRE) was inserted between SV40 poly (A) and heavy-chain sequences to enhance the expression of transgenes, as shown in Figure 1. We used this expression cassette to maintain long-term muscle expression [33].

### Ad-anti-EGFR antibodies expression *in vitro* and *in vivo*

**Figure 2:**
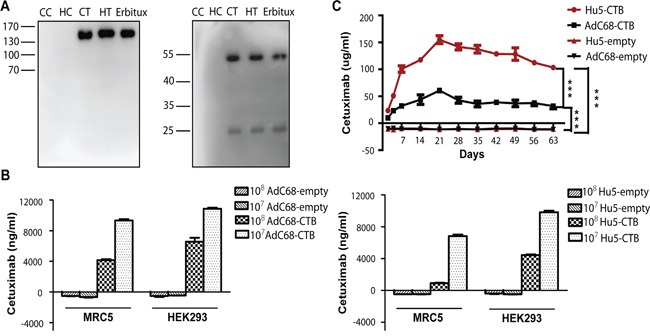
Ad-anti-EGFR antibodies expression *in vivo* and *in vitro* **A.** Western blot analysis of Erbitux and supernatants from AdC68-CTB, and Hu5-CTB-infected HEK293 cells under non-reducing and reducing conditions. CC, AdC68-empty; CT, AdC68-CTB; HC, Hu5-empty; HT, Hu5-CTB. **B.** ELISA analysis of supernatants of AdC68-CTB and Hu5-CTB-infected HEK293 and MRC5 cells. The cells were infected with 1 × 10^8^ vp or 1 × 10^7^ vp AdC68-CTB or Hu5-CTB, respectively. Supernatants were harvested for protein analysis after 3 days. Data are based on three independent experiments. **C.** Anti-EGFR antibody expression level in the serum of immunodeficient mice. Mice were injected with 5 × 10^10^ vp adenovirus into muscles and bled at different time points. The serum anti-EGFR antibody concentrations were determined by sandwich ELISA. Statistical analysis was performed by one-way analysis of variance (ANOVA) with Tukey adjustment. Data are shown as mean ± SEM. *P < 0.05, **P < 0.01 and ***P < 0.001.

In order to characterize the full-length antibody and the heavy and light chain, we performed western blotting. HEK293 cells were infected with AdC68-CTB, Hu5-CTB, AdC68-empty, and Hu5-empty at 10^10^ vp/well for 1 day. The supernatants were collected for reducing or non-reducing SDS-PAGE. Under non-reducing conditions, we detected a specific band of ∼150 kDa, which was the expected size of the dimerized full-length antibody as previously reported [34], while under reducing conditions, approximately 50-kDa heavy chains and 25-kDa light chains were detected (Figure 2A). The results indicated that recombinant adenoviruses AdC68-CTB and Hu5-CTB could express heavy and light chains of the anti-EGFR antibody with a stoichiometric composition. In addition, Ad-anti-EGFR antibodies were correctly assembled as the dimerized full-length antibody.

To further compare the expression of Ad-anti-EGFR antibodies both *in vitro* and *in vivo*, we infected MRC5 and HEK293 cells, and inoculated BALB/c nude mice intramuscularly (i.m.) with adenovirus. The anti-EGFR antibody concentrations in supernatants and mouse sera were determined by sandwich ELISA. As shown in Figure 2B, we observed that the expression *in vitro* was dose-dependent. As expected, anti-EGFR antibody expression in HEK293 cells, which are an E1-complementing cell line, was much higher than that in MRC5 cells. Anti-EGFR antibody in sera of mice treated with AdC68-CTB or Hu5-CTB could be detected 2 days after administration, reached peak at 21 days, and remained at a relative high level for more than 2 months (Figure 2C). Hu5-CTB secreted much more antibodies than AdC68-CTB *in vivo*, although AdC68-CTB expressed a higher level of anti-EGFR antibody *in vitro*. This phenomenon might result from stronger immunogenicity of the Hu5 vector [35].

### Biological activity of Ad-anti-EGFR antibodies

**Figure 3:**
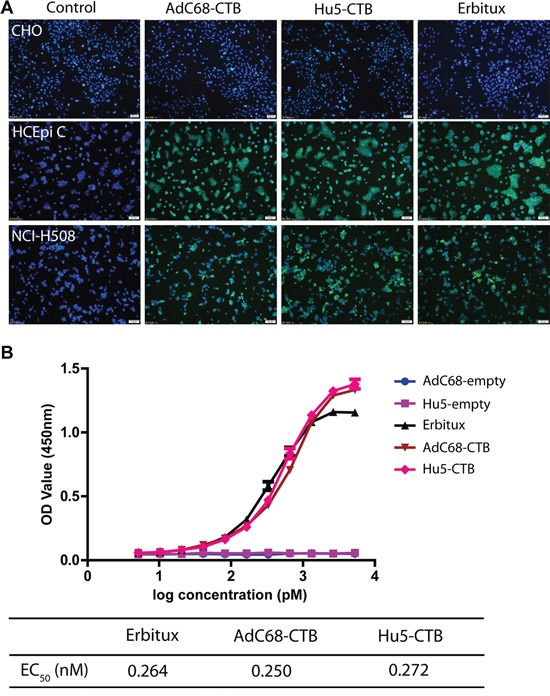
Biological activity of Ad-anti-EGFR antibodies **A.** Specific binding of Ad-anti-EGFR antibodies. Three cell lines CHO (EGFR^−^), HCEpi C (EGFR^+^) and NCI-H508 (EGFR^+^) were selected for binding specificity determination. Ad-anti-EGFR antibodies in mouse serum were used. Erbitux was used as a positive control. Immunofluorescent microscopy analysis was performed for the specific binding of Ad-anti-EGFR antibodies to EGFR. Images are representative of three independent experiments. **B.** Affinity constant of Ad-anti-EGFR antibodies were determined by the EC_50_ values obtained from binding ELISA. The EC_50_ values of anti-EGFR antibody expressed by AdC68-CTB and Hu5-CTB and Erbitux were 0.250 nM, 0.272 nM, and 0.264 nM, respectively. Data are representative of at least three experiments.

To evaluate the binding activity and specificity of Ad-anti-EGFR antibodies, EGFR^+^ cell lines HCEpi C and NCI-H508 and EGFR^−^ cell line CHO were chosen for indirect immunofluorescence. Cetuximab was able to specifically recognize the cell surface EGFR. There was no difference in fluorescence intensity on the surface of EGFR^+^ cells between Erbitux and Ad-anti-EGFR antibodies. However, no FITC fluorescence was observed on the surface of EGFR^−^ cells (Figure 3A). This suggested that Ad-anti-EGFR antibodies exhibited similar specificity to EGFR as Erbitux.

Affinity is the most important target to measure the bioactivity of an antibody; therefore, to further understand the difference in affinity between Ad-anti-EGFR antibodies and Erbitux, we determined the affinity by binding ELISA. The EC_50_ (concentration for half-maximal effect) value was calculated according to the OD value and graphically represented as a function of the analyte concentration. The antibodies expressed by AdC68-CTB or Hu5-CTB and Erbitux bound to EGFR with an EC_50_ of 0.250 nM, 0.272 nM and 0.264 nM, respectively (Figure 3B), which is similar to previously published data [14]. The ELISA data further confirmed that similar affinity could be obtained by Ad-anti-EGFR antibodies.

### Growth inhibition of colorectal cancer cells by Ad-anti-EGFR antibodies

**Figure 4:**
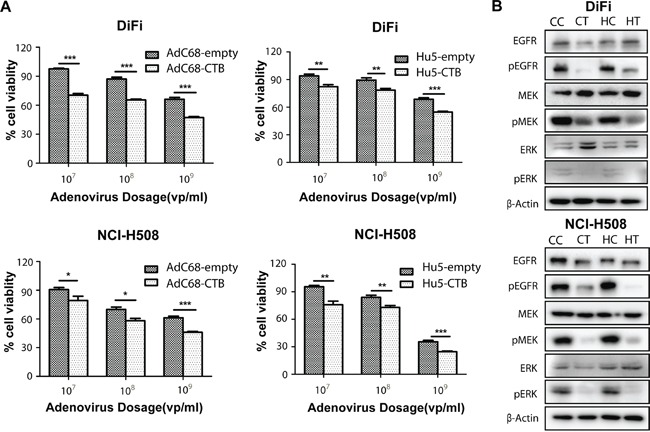
AdC68-CTB and Hu5-CTB inhibits cell proliferation by reduced activation of EGFR, ERK and MEK **A.** NCI-H508 and DiFi cells were grown to a density of 1 × 10^4^ cells/well in 96-well microtiter plates and treated with indicated adenoviruses in doses varying from 10^7^ to 10^9^ vp. After 72 h, an MTT assay was performed to quantify cell viability. Values are expressed as mean ± SEM. Statistical analysis was performed by Student's *t* test (*p < 0.05, **p < 0.01 and ***p < 0.001). Data are based on three independent experiments. **B.** DiFi and NCI-H508 cells were treated with AdC68-CTB or Hu5-CTB for 3 days, and whole-cell lysates were analyzed by Western blot. CC, AdC68-empty; CT, AdC68-CTB; HC, Hu5-empty; HT, Hu5-CTB. Images are representative of at least triplicate experiments.

To evaluate whether Ad-anti-EGFR antibodies can suppress the growth of colorectal cancer cells, we assessed the viability of the colorectal cancer cells infected with AdC68-CTB and Hu5-CTB at different doses. We observed significant dose-dependent growth inhibition of AdC68-CTB or Hu5-CTB in DiFi and NCI-H508 cells (Figure 4A). To gain further insight into the mechanism of how AdC68-CTB and Hu5-CTB suppress the growth of colorectal cancer cells, we evaluated the ability of the recombinant adenovirus to inhibit the EGFR pathway activity. We found that AdC68-CTB and Hu5-CTB inhibited the EGFR pathway activation, as evidenced by the decrease in phosphorylated EGFR, MEK and ERK (Figure 4B). This was in agreement with previous studies, which showed that cetuximab efficiently competed with EGF and TGF-α for binding to EGFR, resulting in the inhibition of receptor activation and downstream signal transduction [1, 13].

### Antitumor activity in xenograft tumor models

**Figure 5:**
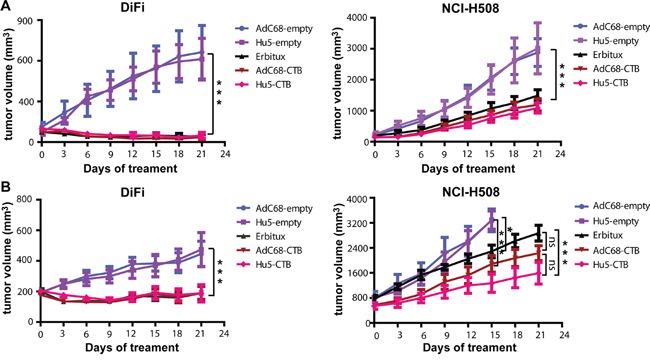
Intramuscular injection of AdC68-CTB or Hu5-CTB suppressed the growth of DiFi and NCI-H508 cells in nude mice DiFi and NCI-H508 cells (1 × 10^7^) were injected s.c. in the right dorsal flank of nude mice. After 6 days (**A.** early stage with smaller size) or 16 days (**B.** late stage with larger size), mice were treated with 5 × 10^10^ vp AdC68-empty, Hu5-empty, AdC68-CTB, Hu5-CTB or cetuximab (20 mg/kg, twice weekly). Development of tumors (mean ± SEM) was monitored using calipers every 3 days. Statistical analysis was performed by two-way ANOVA (*p < 0.05, **p < 0.01 and ***p < 0.001).

We set out to identify the antitumor activity of AdC68-CTB and Hu5-CTB in human colorectal cancer xenograft tumor models by early or late therapeutic strategies. The three treatment groups (AdC68-CTB, Hu5-CTB and Erbitux group) presented a remarkable reduction in tumor volume relative to the controls (AdC68-empty and Hu5-empty group) in both strategies (Figure 5A, 5B). AdC68-CTB, Hu5-CTB and Erbitux exerted similar anti-tumor activities in all treatment groups except the late treatment group of NCI-H508 xenografts. In this group, Hu5-CTB showed stronger inhibition than Erbitux (1059±134.7 cm^2^ vs. 1882±254.8 cm^2^, *p* < 0.001) (Figure 5B). Notably, in the early treatment group of DiFi xenografts, AdC68-CTB, Hu5-CTB and Erbitux caused significant tumor regression (Figure 5A). Specifically, tumors regressed completely in 50% of AdC68-CTB-treated mice (n=10), 50% of Hu5-CTB-treated mice (n=10), and 40% of Erbitux-treated mice (n=10). In addition, no evidence of toxicity was observed in all the animal experiments, which was monitored by the body weight (data not shown). It is evident that earlier treatment confers better clinical outcomes.

**Figure 6:**
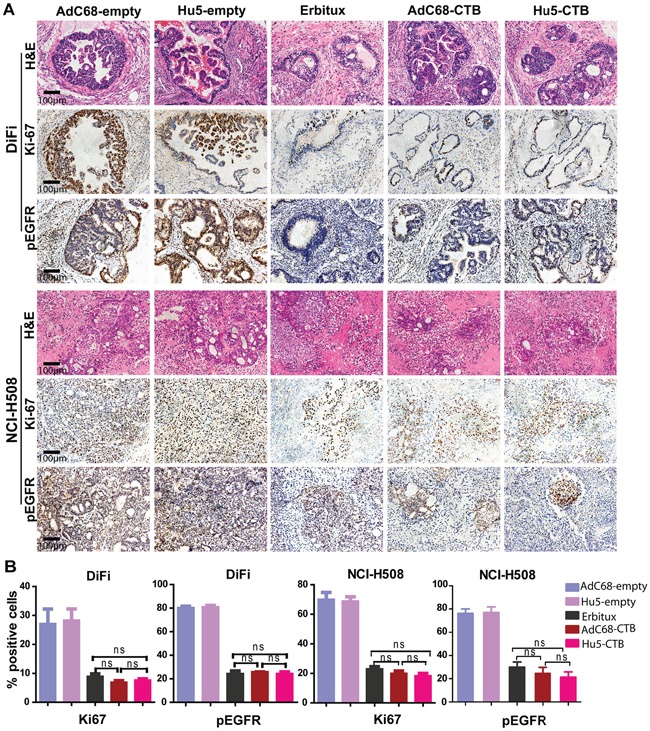
Ki-67 and pEGFR were reduced after treatment in early therapeutic strategy Mice bearing colorectal carcinoma were treated with 5 × 10^10^ vp AdC68-empty, Hu5-empty, AdC68-CTB, Hu5-CTB or cetuximab (20 mg/kg, twice weekly) for 21 days, and tumor sections (n=10 per treatment group, n=6 for controls) were stained with Ki-67 and pEGFR. **A.** IHC photographs were representative fields from animals in each group. Scale bars, 100μm. **B.** The graphs shown the percentages of positively stained cells (control groups vs. treatment groups *p* < 0.001, except for Ki-67 in DiFi xenograft tumors *p* < 0.01). To semiquantify the Ki-67 and pEGFR-positive areas, three fields from each sample were randomly selected. Values are expressed as mean ± SEM. Statistical significance was assessed by one-way ANOVA with Tukey adjustment: *p < 0.05; **p < 0.01; ***p < 0.001; ns (not significant).

**Figure 7:**
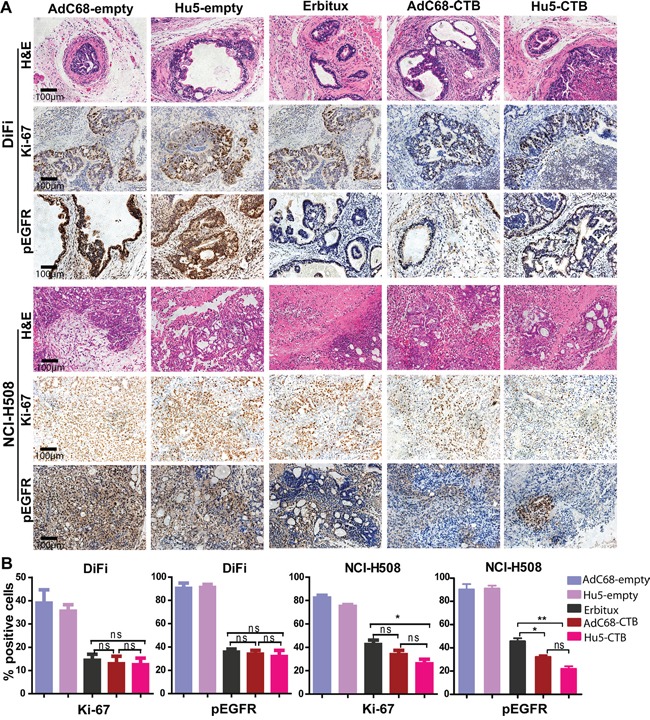
Immunohistochemical analysis of xenograft tumors for expression of Ki-67 and pEGFR in late therapeutic strategy Tumors stained and quantified as in Fig. 6. Treated animals exhibited reduced Ki-67 and pEGFR staining compared with control cohorts (*p* < 0.001, except for Ki-67 in DiFi xenograft tumors *p* < 0.01). **A.** Representative images collected from control (n=6) and treated (n=10) mice. Scale bars, 100μm. **B.** The graphs of percentage area positive for Ki-67 and pEGFR in tumor specimens. Values are expressed as mean ± SEM. Statistical significance was assessed by one-way ANOVA with Tukey adjustment: *p < 0.05; **p < 0.01; ***p < 0.001; ns (not significant).

Furthermore, we performed immunohistochemical staining for pEGFR and Ki-67 to prove the treatment effects on tumor cells proliferation. Ki-67, a tumor growth marker for proliferation, is associated with tumor aggressiveness or progression in numerous malignancies [36]. In the early therapeutic strategy, we observed the reduction in pEGFR and Ki-67 staining after treatment with AdC68-CTB, Hu5-CTB, or Erbitux (Figure 6A). There was no significant difference among all treatment groups (Figure 6B). As to late therapeutic strategy, AdC68-CTB, Hu5-CTB, or Erbitux treatment down-regulated pEGFR and Ki-67 (Figure 7A). Notably, pEGFR were inhibited more effectively by AdC68-CTB (32.3±2.62%) or Hu5-CTB (22.0±3.80%) compared with Erbitux (45.8±4.32%) (Figure 7B). Hu5-CTB significantly lowered Ki-67 with Erbitux (25.97±6.84% vs. 42.34±6.50%, *p* < 0.05). We hypothesized that the treatment effect would be more pronounced with higher cetuximab dose. Positive correlation between the tumor volume and standard immunohistochemistry was observed.

## DISCUSSION

Here, we developed an anticancer strategy, which could induce robust and persistent expression of anti-EGFR antibody with one single administration of a recombinant adenovirus. Ad-anti-EGFR inhibited cancer cell growth by reducing activation of EGFR, ERK and MEK *in vitro*. On the other hand, administration of Ad-anti-EGFR could produce a bioactive anti-EGFR antibody that suppressed tumor growth in mice. In summary, the monoclonal antibody (mAb) expression system described in this study can be effectively applied to therapeutic regimens in which stable generation of mAb is desired.

Expression of antibodies *in vivo* is an advantageous approach that can provide a high concentration of pure antibodies over a long period at a low cost. Fang *et al.* developed a furin/2A self-processing sequence that allowed balanced co-expression of heavy and light chains by an Adeno-associated virus (AAV) [25]. However, the use of AAV as an expression vector is limited by the packaging capacity, difficult manufacturing processes and cost [37, 38]. On the other hand, high-titers of adenoviruses can be readily prepared. The E3 region of adenovirus genome is non-essential for viral growth [39]. Adenoviruses with a deletion of both E1 and E3 genes are replication defective and can accommodate up to 8 kb of foreign DNA, which can be pursued as ideal expression vectors for mAb. In this study, we generated E1/E3-deleted adenovirus vectors that encoded the heavy and light chains. The two domains were linked by furin/2A and under the control of the powerful CASI promoter. Notably, mAb expression from this system is rapid and maintains at a high level for >2 months after only a single administration in immunodeficient mice.

There is one concern however, that long-lived transgene expression could be out of control and induce adverse events in patients, which will require treatment discontinuation. AAV can integrate into the patient genome and appears to provide a long-lived transgene expression. Adenovirus vectors, on the other hand, do not integrate and only transiently express the transgene. Furthermore, the mAb expression system described here can be readily switched on and off, thus offering an alternative approach for producing sufficient therapeutic antibodies *in vivo*. Although the antibody level in Hu5-CTB treated mice sera was higher than AdC68-CTB group, we failed to observe significant improvement in therapeutic effect. We therefore reasoned that the anti-EGFR antibody expression induced by AdC68-CTB was already sufficient for therapy. Plasma levels in AdC68-CTB treated mice was above 30μg/ml, which was within effective therapeutic limits (3–30 μg/ml) [40]. Considering the low prevalence rate of AdC68, we believe AdC68 may be a better choice.

In our studies, one single administration of AdC68-CTB achieved the similar efficacy with Erbitux (twice per week). Intravenous infusion of Erbitux takes more than 2 hours in clinical, which may cause infusion reactions and even death [41]. On the other hand, intramuscular injection, adopted by AdC68-CTB, is safer and less time-consuming. Moreover, lower cost of adenovirus can confer patients more benefits. However, adenoviruses require storage at low temperatures (−70°C), new formulations to render them thermostable are urgently needed. Generally, AdC68-CTB may be an alternative agent to Erbitux.

In conclusion, our data proved the feasibility of AdC68-CTB as a potential antitumor agent. AdC68-CTB could induce inhibition of cancer cell growth, which was in line with published cetuximab treatment effect [42]. Next, increase in the efficiency of AdC68-CTB will be the focus of future work. We will develop an oncolytic AdC68-CTB targeted to tumor tissue.

## MATERIALS AND METHODS

### Cell lines and antibodies

The human colorectal cancer cell lines DiFi, NCI-H508, as well as the human embryonic kidney cell line HEK293 were obtained from the American Type Culture Collection (ATCC, Manassas, VA). The normal human fibroblast cell line MRC5 and human corneal epithelial cell lines HCEpi C were purchased from Shanghai Institutes for Biological Sciences (Shanghai, China). The Chinese hamster ovary cell line CHO was kindly gifted by Zhong Huang, from Institut Pasteur of Shanghai, Chinese Academy of Sciences, Shanghai, China. The cells were cultured according to the supplier's instructions.

The commercial antibodies used were: human lgG, H&L (abcam, ab6759-HRP), human Kappa (Southern Biotech, 2060-01), human lgG Fc (abcam, ab99765-HRP), Ki-67 (abcam, ab66155), rabbit IgG(abcam, ab64256-biotin), human lgG (Santa Cruz, sc-2456-FITC), EGFR (CST, #4267), pEGFR (abcam, ab32430), MEK, pMEK (CST, #9120), Erk1/2 (CST, #9102), pErk1/2 (CST, #9106), and β-actin (Sigma-Aldrich, A5441).

### Recombinant adenovirus production

To create the recombinant adenovirus AdC68-CTB and Hu5-CTB, we synthesized the vector pUC57-lgG (GenScript, Nanjin, China), which contains the full-length heavy and light chains of cetuximab (Patent WO 2008/083949 A2), and the vector pUC57-CASI, which includes the CASI promoter (Patent US 2012/023233 A1). To generate pUC57-CTB plasmid, an *AvrII-ClaI* fragment, containing the full-length heavy and light chains of cetuximab, was excised from the pUC57-lgG plasmid, and sub-cloned into the corresponding sites of pUC57-CASI.

pUC57-CTB was digested with *I-CeuI* and *PI-SceI*, and then inserted into the *I-CeuI-PI-SceI*-digested adenovirus vector based on Hu5, or AdC68. Both vectors were rendered incompetent to replicate by deletion of the E1 and E3 viral genes to create two adenoviral plasmids, pAdC68-CTB and pHu5-CTB. These plasmids were linearized by digestion with *PacI* and transfected into HEK293 cells to generate recombinant adenoviruses, AdC68-CTB and Hu5-CTB. The adenoviruses were then amplified in HEK293 cells and purified by CsCl gradient centrifugation.

### Western blot

HEK293, DiFi and NCI-H508 cells were plated onto 6-well plates, and after incubation for 24 h, infected with AdC68-CTB or Hu5-CTB, at 10^10^ vp per well. AdC68-empty and Hu5-empty were used as controls. HEK293 cell culture supernatants were harvested after 24 h. Supernatants and Erbitux were separated on 10% SDS-PAGE under reducing or non-reducing conditions. The DiFi and NCI-H508 cells were lysed after 72 h, in RIPA lysis buffer (Beyotime, Shanghai, China) supplemented with protease and phosphatase inhibitors (Roche, Indianapolis, IN) and separated by 10% SDS-PAGE under reducing conditions. The separated proteins were transferred to nitrocellulose membranes, then, blocked with 5% non-fat milk in PBS. The primary antibodies, rabbit anti-human EGFR, pEGFR(Y1068), MEK1/2, pMEK1/2, Erk1/2 and pErk1/2 were used at a dilution of 1:1000 and the secondary antibodies, HRP-conjugated mouse anti-human lgG (H&L), anti-rabbit lgG (H&L) were used at a dilution of 1:5000.

### ELISA

For anti-EGFR antibody expression *in vitro*, MRC5 and HEK293 cells were seeded on 6-well plates, incubated for 24h, and then infected with AdC68-CTB or Hu5-CTB. AdC68-empty and Hu5-empty were used as controls. Supernatants were harvested at days 3. For *in vivo* expression, 24 healthy female BALB/c nude mice, 6-8 weeks of age, were randomly divided into 4 groups (AdC68-CTB, Hu5-CTB, AdC68-empty and Hu5-empty), of 6 mice each, which were inoculated i.m., with adenovirus, at 5 × 10^10^ vp/mouse in 100 μl. Each mouse was bled at different time points following adenovirus administration, from day 2 to 63.

Anti-EGFR antibody produced *in vitro* or *in vivo*, at different time points, were determined by sandwich ELISA. The anti-human lgG Kappa (70 ng/well) was coated onto the ELISA plate which then was blocked with 5% skim milk. After adding diluted cell supernatants or mouse serum, HRP-conjugated mouse anti-human lgG Fc (1:10000) was added. A standard curve was generated using Erbitux. The absorbance was measured at 450 nm using a microtiter plate reader (Thermo Scientific, Waltham, MA). The specificity and affinity of the anti-EGFR antibody were determined by binding ELISA, which is the same as sandwich ELISA, except that the plate was coated with EGFR (60 ng/well, abcam) and the mouse serum and Erbitux were diluted from 80 ng/well to 0.078125 ng/well.

### Indirect fluorescence assay

EGFR^+^ cell lines, including HCEpi C and NCI-H508, and EGFR^−^ cell line CHO, were seeded onto 24-well plates. After 24 hours of incubation, cells were fixed with 4% paraformaldehyde and then incubated for 2 h with cell supernatants from adenovirus infected HEK293 cells. Erbitux was used as a positive control. The secondary antibody, anti-human lgG conjugated with FITC, was applied at a dilution of 1:200 for 1 h. The cells were then stained with DAPI for 5 min in the dark. Images were acquired by the Olympus fluorescence microscope (10× objective).

### MTT assay

DiFi and NCI-H508 cells were seeded at 10^4^/well in 96-well plates and incubated overnight. The AdC68-CTB, Hu5-CTB, AdC68-empty, and Hu5-empty at various multiplicities of infection (10^7^−10^9^ vp) were added to each cell line. After incubation for 72 h, the cells were treated with 0.5 mg/ml semi-automatic 3-(4,5-dimethyl-thiazol-2-yl)-2,5-Diphenyl-tetrazolium bromide (MTT, Sigma) for 4 h, and then dissolved in 150 μl dimethylsulfoxide (DMSO) for 20 min and the absorbance at 490 nm was measured using a microplate reader (Thermo Scientific, Waltham, MA). Averages were calculated from at least three independent experiments.

### Xenograft cancer model

Six to eight-weeks-old female BALB/c nude mice were obtained from Shanghai Laboratory Animal Center (Shanghai, China) and housed under specific pathogen-free conditions. All animal experiments were approved by the Institutional Animal Care and Use Committee, at the Institut Pasteur of Shanghai. Mice were injected subcutaneously (s.c.) on the right dorsal flank with 10^7^ DiFi or NCI-H508 cells, together with a matrix gel (1:1, BD Biosciences, San Jose, CA). Gene therapy in xenograft mice was performed in two groups: the early and late stage. For early stage groups, treatment was initiated at 6 days after cell inoculation, whereas treatment of the late stage groups was implemented at 16 days. The mice were randomly distributed into 5 groups: AdC68-CTB, Hu5-CTB, Erbitux, AdC68-empty, and Hu5-empty. The treatment groups received an intramuscular injection of 5 × 10^10^ vp AdC68-CTB (n = 10), Hu5-CTB (n = 10), or 20 mg/kg Erbitux (n = 10) into the peritoneum and the negative control groups were injected with 5 × 10^10^ vp AdC68-empty (n = 6), Hu5-empty (n = 6) into muscles. Adenovirus-injected mice received a single dose, while Erbitux was given twice weekly for 3 total weeks of treatment. This does of 20mg/kg and regimen are based on previous studies [43]. Tumor volumes were measured by digital calipers every three days, following the formula of length × width^2^ × 0.5. Animals were euthanatized when the tumor volume exceeded 3,000 mm^3^.

### Immunohistochemical analysis

After 21 days of treatment, tumors from all groups were subsequently embedded in paraffin. The specimens were cut into 5 mm thin sections, which were stained with hematoxylin and eosin (H&E). For immunohistochemistry, primary antibodies were as follows: rabbit monoclonal anti-pEGFR antibody and rabbit monoclonal anti-Ki-67 antibody. Briefly, after deparaffinization and rehydration, sections were treated with citrate buffer solution (PH 6.0) in a water bath at 99°C and cooled for 20 min for antigen retrieval. Sections were then blocked with 10% serum, 3% BSA in PBS for 1 h at room temperature and then incubated with primary antibodies overnight at 4°C. The secondary antibody used was biotinylated goat anti-rabbit IgG. Images were acquired by the Olympus fluorescence microscope (20× objective).

### Statistical analysis

The values were expressed as mean ± SEM. Differences between groups were analyzed by Student's *t* test, one-way analysis of variance (ANOVA) with Tukey adjustment, and two-way ANOVA. Prism 5 Software (GraphPad, La Jolla, CA) was used to draw the graphs and perform statistical analysis. For all tests, P < 0.05 was considered significant.
